# Cathepsin S activation contributes to elevated CX3CL1 (fractalkine) levels in tears of a Sjögren’s syndrome murine model

**DOI:** 10.1038/s41598-020-58337-4

**Published:** 2020-01-29

**Authors:** Runzhong Fu, Hao Guo, Srikanth Janga, Minchang Choi, Wannita Klinngam, Maria C. Edman, Sarah F. Hamm-Alvarez

**Affiliations:** 1 Department of Pharmacology and Pharmaceutical Sciences, School of Pharmacy, Roski Eye Institute, Keck School of Medicine, University of Southern California, Los Angeles, CA 90033 USA; 2 Department of Ophthalmology, Roski Eye Institute, Keck School of Medicine, University of Southern California, Los Angeles, CA 90033 USA

**Keywords:** Lacrimal apparatus diseases, Autoimmune diseases

## Abstract

Autoimmune dacryoadenitis and altered lacrimal gland (LG) secretion are features of Sjögren’s syndrome (SS). Activity of cathepsin S (CTSS), a cysteine protease, is significantly and specifically increased in SS patient tears. The soluble chemokine, CX3CL1 (fractalkine), is cleaved from membrane-bound CX3CL1 by proteases including CTSS. We show that CX3CL1 is significantly elevated by 2.5-fold in tears (p = 0.0116) and 1.4-fold in LG acinar cells (LGAC)(p = 0.0026) from male NOD mice, a model of autoimmune dacryoadenitis in SS, relative to BALB/c controls. Primary mouse LGAC and human corneal epithelial cells (HCE-T cells) exposed to interferon-gamma, a cytokine elevated in SS, showed up to 9.6-fold (p ≤ 0.0001) and 25-fold (p ≤ 0.0001) increases in *CX3CL1* gene expression, and 1.9-fold (p = 0.0005) and 196-fold (p ≤ 0.0001) increases in CX3CL1 protein expression, respectively. Moreover, exposure of HCE-T cells to recombinant human CTSS at activity equivalent to that in SS patient tears increased cellular CX3CL1 gene and protein expression by 2.8-fold (p = 0.0021) and 5.1-fold (p ≤ 0.0001), while increasing CX3CL1 in culture medium by 5.8-fold (p ≤ 0.0001). Flow cytometry demonstrated a 4.5-fold increase in CX3CR1-expressing immune cells (p ≤ 0.0001), including increased T-cells and macrophages, in LG from NOD mice relative to BALB/c. CTSS-mediated induction/cleavage of CX3CL1 may contribute to ocular surface and LG inflammation in SS.

## Introduction

Sjögren’s syndrome (SS) is a systemic autoimmune disease associated with lymphocytic infiltration of lacrimal glands (LG) and salivary glands (SG), associated with dacryoadenitis and sialoadenitis, respectively^1^. SS patients develop associated complications including reduced tear and saliva production, blurred vision, corneal damage, dental cavities and oral thrush^1^. Inflammation in the LG promotes release of pro-inflammatory cytokines to the ocular surface, which can further compromise tear secretion by disruption of corneal sensory and efferent nerve responses^2,3^. This further reduces tear flow and alters tear composition, resulting in pro-inflammatory and proteolytic tears^4^ which may elicit apoptosis and autophagy to further damage the ocular surface^5^. Reduced tear volume, tear film instability and ocular surface inflammation all contribute to the reduced visual acuity and increased patient discomfort associated with dry eye symptoms in SS^2,6^.

The male non-obese diabetic mouse (NOD) is commonly used as a model of the autoimmune dacryoadenitis and ocular surface inflammation characteristic of SS. While the ocular manifestations of SS spontaneously develop in the males from 8–10 weeks of age, the female mouse develops a later autoimmune sialoadenitis from 14–16 weeks of age^7^. The male NOD mice share many ocular surface system manifestations with SS patients including lymphocytic infiltration of the LG^7,8^, reduced tear flow^7–10^, generation of a proteolytic tear film^7,9,10^, altered distribution and expression of Rab3D^9,11^, reduced myoepithelial cells^12^, loss of extracellular matrix^8^ and elevated cytokines in LG and tears^13^.

Cathepsin S (CTSS) activity is markedly elevated in the LG and tears of male NOD mice^14^. This lysosomal cysteine protease has diverse physiological functions including degradation of extracellular matrix, processing of major histocompatibility complex II (MHC II) for antigen presentation, and protein catabolism^15,16^. Due to its distinct role in antigen presentation, CTSS is implicated in autoimmune diseases including rheumatoid arthritis (RA), systemic lupus erythematosus and multiple sclerosis^17^. Following studies in the male NOD model^14^, CTSS activity was demonstrated as significantly elevated in SS patient tears relative to tears of patients with non-SS dry eye or other autoimmune diseases^10^. *In vitro* studies suggest that elevated tear CTSS may affect ocular surface homeostasis, since it can induce expression and secretion of pro-inflammatory cytokines and matrix metallopeptidase 9 (MMP-9) in a corneal cell line^18^. In the male NOD mouse, CTSS activity and protein are increased not only in tears but also in the protein-secreting acinar cells of the LG (LGAC), suggesting additional actions^9,13^. With knowledge of the relationship of other protease-sensitive pathways to inflammation, we sought out potential targets of CTSS that might be implicated in the etiology of SS.

CX3CL1 (fractalkine), is the only member of the subclass of CX3C chemokines^19^. Existing in two forms, membrane-bound and soluble, it is one of only two chemokines to exist in both forms^19,20^. Synthesised as the membrane-bound form, it is cleaved to the soluble form through proteases including metallopeptidase 10 (ADAM-10)^21^, metallopeptidase 17 (ADAM-17)^22^, MMP-9^23^ and CTSS^24^. Some studies have suggested that organ-specific cleavage products resulting from different protease cleavage may have distinct actions^23^. Membrane-bound CX3CL1 has a chemokine domain, a mucin-like stalk, a transmembrane α-helix and a cytoplasmic tail, while soluble CX3CL1 contains only the chemokine domain and mucin-like stalk^20^. Both membrane-bound and soluble CX3CL1 recognise the G protein-coupled receptor, CX3CR1, abundantly expressed on immune cells including natural killer (NK) cells, dendritic cells, T-cells, monocytes and macrophages^25^. Both forms of CX3CL1 are implicated in adhesion and chemoattraction^26^. CX3CL1 is frequently expressed on vascular endothelium, where it facilitates leukocyte extravasation by capturing CX3CR1-expressing cells from the blood, inducing transmigration and recruitment to tissue^27^.

CX3CL1 has been implicated in systemic lupus erythematosus^28^ and rheumatoid arthritis^29^. Intriguingly, CX3CL1 is also elevated in the serum and SG of primary SS patients^30^. A previous study also showed that CX3CL1 expression was elevated in LG of thymectomised NFS/sld mice, another model of SS-associated dacryoadenitis^31^. We hypothesised that the elevated CTSS in tears and LG in SS might participate in the generation of soluble CX3CL1, contributing to pathogenesis in the lacrimal functional unit.

IL-1, IL-6, IL-8, TNF-α, interferon-γ (IFN-γ) and IL-17 are all increased in SS patient tears^32–35^. *In vitro* exposure of LG acinar cells (LGAC) to IFN-γ evokes some of the same SS-associated changes characteristic of disease, including altered organisation of Rab3D and induction of antigen presentation^13^. Since IFN-γ also induces the expression of membrane-bound CX3CL1 and reinforces its functions^11^, we used *in vitro* exposure to IFN-γ to induce CTSS and CX3CL1 expression to further study their interactions^36^.

In this study, we investigated the relationships between CX3CL1, CX3CR1 and elevated CTSS *in vivo* in the male NOD mouse and *in vitro* in primary LGAC and corneal epithelial cells primed with IFN-γ to induce disease-like changes prior to treatments with CTSS. Our results show that 1) CX3CL1 is elevated in tears, LG and corneas of male NOD mice; 2) IFN-γ induces CX3CL1 and CTSS expression in LGAC and corneal epithelial cells; 3) CTSS induces CX3CL1 and increases its release into culture medium in corneal epithelial cells; and 4) CX3CR1+ immune cells are elevated in NOD mouse LG. These results suggest a relationship between elevated CTSS activity, CX3CL1 induction and shedding, and immune cell homing that may play a role in SS pathogenesis.

## Results

### CX3CL1 (fractalkine) is elevated in tears of NOD mice

**Figure 1:**
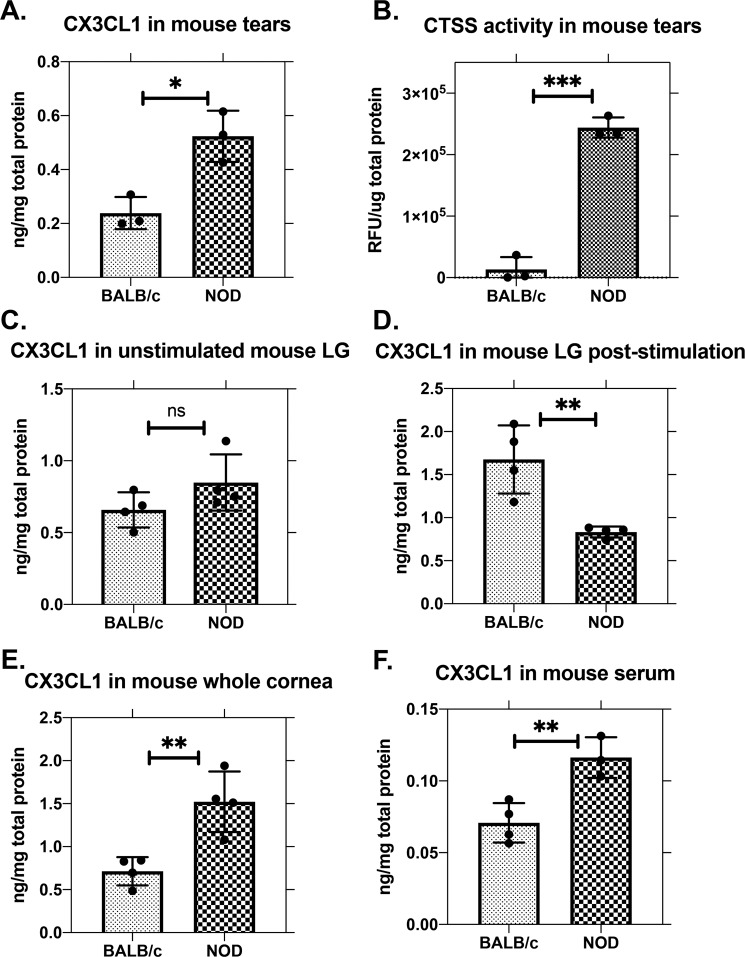
CX3CL1 protein and CTSS activity are elevated in NOD mouse tears relative to BALB/c mouse tears. (**A**) Soluble CX3CL1 (fractalkine) was significantly increased in stimulated tears from NOD mice compared to BALB/c mice (p = 0.0116, N = 3). (**B**) CTSS activity was significantly increased in the same reflex tears of NOD mice relative to BALB/c mice used for CX3CL1 analysis in (A). (p = 0.0001, N = 3). (**C**) CX3CL1 levels in NOD and BALB/c mouse LG in the absence of topical carbachol (CCh) stimulation were not significantly different (p = 0.1501, N = 4). (**D**) CX3CL1 levels in NOD mouse LG were significantly reduced relative to BALB/c mouse LG levels by topical CCh stimulation (p = 0.0057, N = 4). (**E**) CX3CL1 was significantly increased in the corneal buttons from NOD mice relative to BALB/c (p = 0.0059, N = 4). (**F**) CX3CL1 was significantly elevated in NOD mouse serum relative to serum from BALB/c mice p = 0.0080, N = 4). In (A,B,E), each N value consisted of tears or corneal buttons from 2 mice, pooled for 1 sample, while in (C,D,F), N = mouse number. Panels (A,C,D,E,F) were measured using ELISA, while panel (B) was measured with a commercial CTSS fluorescence activity kit. All data are presented as mean ± SD.

To determine whether CX3CL1 was altered in tears of diseased male NOD mice versus healthy age-matched male BALB/c mice, we measured CX3CL1 abundance in tears and tissue from these models. A significant increase in CX3CL1 was observed in tears of NOD mice (Fig. 1A), correlated with its decrease in LG after carbachol (CCh)-induced tear stimulation (Fig. 1D). In unstimulated LG, CX3CL1 abundance was not significantly different between strains (Fig. 1C). CTSS activity was measured in the same tear samples in Fig. 1A, revealing a marked increase in NOD mice (Fig. 1B) paralleling increased CX3CL1. Membrane-bound CX3CL1 has multiple cysteine protease cleavage sites and could undergo cleavage by CTSS, consistent with increased soluble CX3CL1 in NOD mouse tears. CX3CL1 was found to be significantly increased in corneal buttons of NOD mice (Fig. 1E), which was associated with upregulated gene expression in the cornea (Supplementary Fig. S1). CX3CL1 was also significantly elevated in NOD mouse serum relative to BALB/c mice (Fig. 1F), consistent with reports in SS patients^30^.

### CX3CL1 and CTSS are increased in NOD mouse LGAC

**Figure 2:**
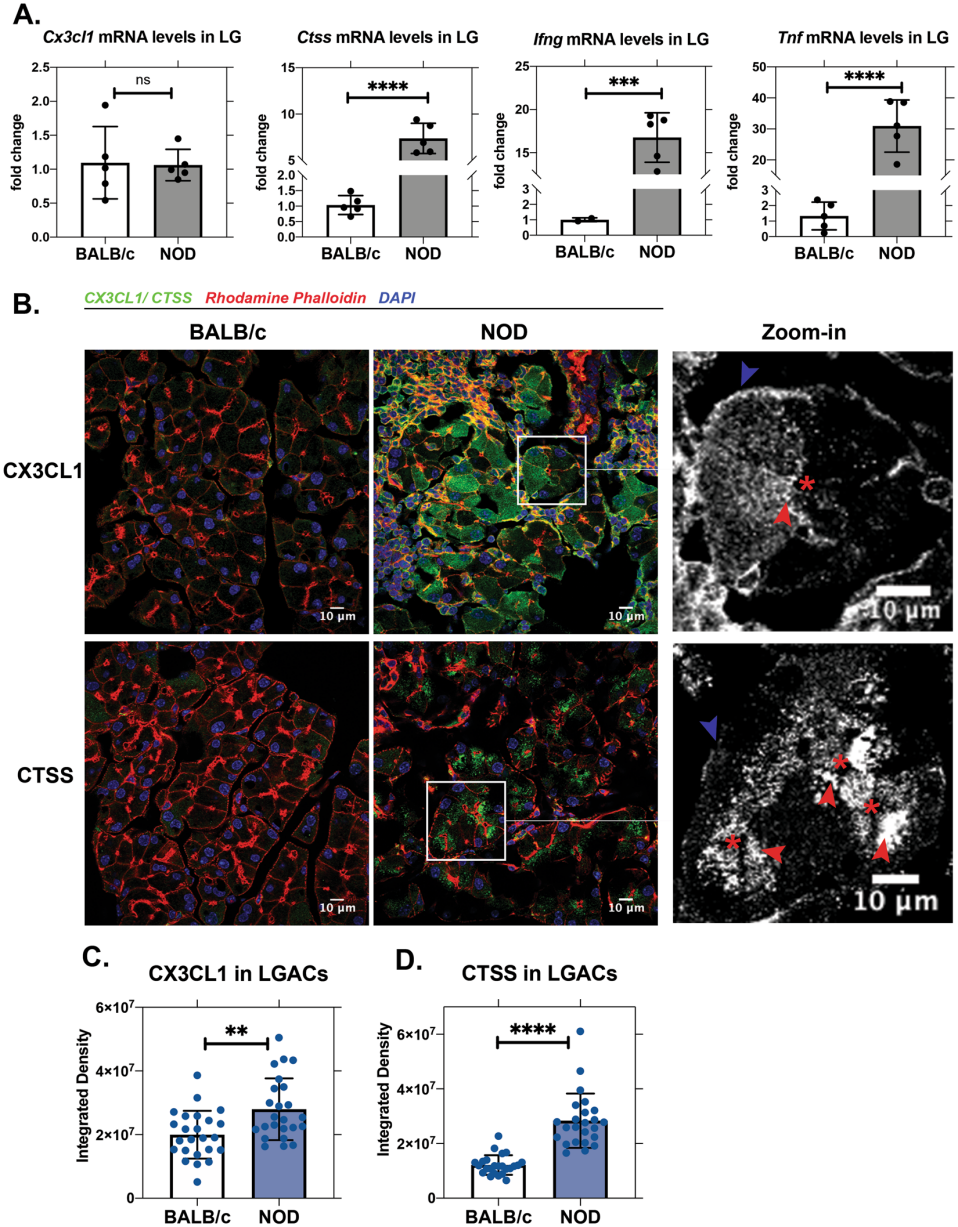
CX3CL1 and CTSS expression are elevated in NOD mouse LG relative to BALB/c. (**A**) Gene expression of *Ctss* and pro-inflammatory cytokines were significantly elevated in LG from NOD mice relative to BALB/c mice, but no difference in *Cx3cl1* gene expression was observed (*Cx3cl1*: p = 0.901, *Ctss*: p ≤ 0.0001, *Ifng*: p = 0.007, *Tnf*: p ≤ 0.0001, N = 5). *Ifng* gene expression was undetectable in 3 BALB/c mice. (**B**) Indirect immunofluorescence revealed increased levels of CX3CL1 and CTSS protein in acinar cells in NOD mouse LG relative to levels in BALB/c. In zoomed images of NOD LGAC, apical and basolateral CX3CL1/CTSS are marked with red and blue arrowheads, respectively, and the lumen is marked with an asterisk. Image quantification is shown in (**C)** with p = 0.0026 for CX3CL1 and (**D**) with p ≤ 0.0001 for CTSS, respectively (N = 4). For immunofluorescence analysis, N = mice per group while sections from each mouse LG were evaluated in 6 ROI per sample, each of which is represented as points on the graph. All data are presented as mean ± SD.

Many proteins present in aqueous tears are produced and secreted by LGAC. To elucidate the source of increased CX3CL1 in NOD mouse tears, we compared the expression of *Cx3cl1* and *Ctss* in NOD and BALB/c LG. Gene expression of *Ctss*, together with the pro-inflammatory cytokines, *Ifng* and *Tnf*, were significantly elevated in NOD mouse LG (Fig. 2A) consistent with previous reports^13^. No changes in *Cx3cl1* gene expression were observed (Fig. 2A). The percentage of LGAC relative to other cells in the LG decreases as lymphocytes extensively infiltrate the gland in disease. With this knowledge and the observation that CX3CL1 protein was decreased in stimulated LG from NOD mice (Fig. 1D), we analysed its abundance specifically in LGAC using quantitative immunofluorescence. Supplementary Fig. S2 shows a section across the entire LG indicating increased CX3CL1 protein in NOD LG compared to BALB/c. Quantification of proteins distributed in LGAC (Fig. 2B) with the corrected total cell fluorescence (CTCF) method^37^ revealed that CX3CL1 (Fig. 2B,C) and CTSS protein levels (Fig. 2B,D) were significantly elevated in LGAC from NOD mice compared to BALB/c. Magnified images of LGAC (Fig. 2B) demonstrated vesicular basolateral and apical enrichment of CX3CL1 and CTSS, consistent with possible secretion/shedding at either plasma membrane domain.

### *In vitro* IFN-γ induces CX3CL1 in BALB/c LGAC

**Figure 3:**
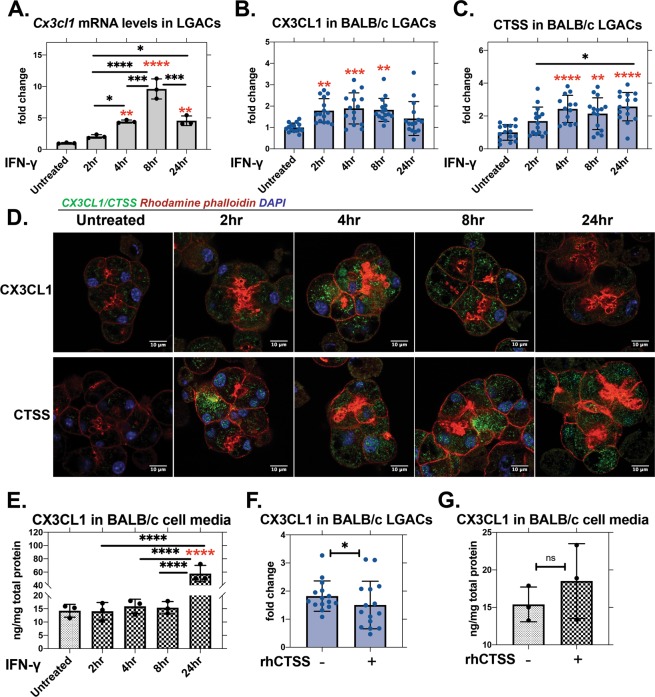
Recombinant mouse IFN-γ (200 U/ml) induces *Cx3cl1* gene expression and elevates CX3CL1 protein in culture medium of cultured LGAC from BALB/c mice. **(A)** Gene expression of *Cx3cl1* in cultured LGAC from BALB/c mice was increased by IFN-γ treatment. Relative to untreated (red asterisk): 4 hr, p = 0.0014; 8 hr, p ≤ 0.0001; 24 hr, p = 0.0011. Between groups (black asterisk): 2 hr vs. 4 hr, p = 0.0319; 2 hr vs. 8 hr, p ≤ 0.0001; 2 hr vs. 24 hr, p = 0.0238; 4 hr vs. 8 hr p = 0.0001; 8 hr vs. 24 hr, p = 0.0001 (N = 3). (**B**) CX3CL1 protein in LGAC as quantified by immunofluorescence intensity from confocal microscopy images typical of those in panel (E), showed significant increases in CX3CL1 from 2–8 hr of IFN-γ incubation. Relative to untreated: 2 hr, p = 0.0025; 4 hr, p = 0.0005; 8 hr, p = 0.0014 (N = 3). (**C**) CTSS in LGAC as quantified from images typical of those in panel (E), showed increased CTSS by 4 hr of IFN-γ sustained throughout the treatment time. Relative to untreated (red asterisk): 4 hr, p ≤ 0.0001; 8 hr, p = 0.0011; 24 hr, p ≤ 0.0001. Comparison between different IFN-γ treatment times showed (black asterisk): 2 hr vs. 24 hr, p = 0.0334 (N = 3). (**D**) Confocal fluorescence microscopy of LGAC shows that CX3CL1 and CTSS image intensities change with time of exposure to IFN-γ. Green, CX3CL1; red, F-actin; blue, DAPI staining. (**E**) Soluble CX3CL1 detected by ELISA in culture media was significantly elevated by IFN-γ treatment for 24 hr. Relative to untreated (red asterisk): 24 hr, p ≤ 0.0001. Comparison between different IFN-γ exposure times (black asterisk): 2 hr vs. 24 hr, p ≤ 0.0001; 4 hr vs. 24 hr, p ≤ 0.0001; 8 hr vs. 24 hr; p ≤ 0.0001 (N = 3). (**F**) Membrane-bound CX3CL1 analysed by immunofluorescence was significantly reduced with 2 hr incubation with active recombinant CTSS (20,000 RFU/treatment) in LGAC after pretreatment with IFN-γ for 8 hr. Ratio paired t-test, p = 0.0322. (**G**) In medium collected from the experiments in panel (F), soluble CX3CL1 was not significantly increased by additional CTSS treatment in the presence of IFN-γ. Paired t-test: p = 0.3503, From panels (A–E), comparisons between treated and untreated cells were tested with one-way ANOVA Dunnett’s multiple comparison test, demonstrated with red asterisk. Comparison between treatment groups was analysed with one-way ANOVA with Tukey’s multiple comparison test, demonstrated with black asterisk. N = preparations, Each preparation included LGAC isolated from 10 BALB/c mice. For confocal microscopy image quantification, 5 ROIs were obtained from each preparation and represented as points on the image. All data are presented as mean ± SD.

Previously, we demonstrated that *in vitro* IFN-γ treatment of BALB/c mouse LGAC induces changes comparable to those seen in NOD mice LG. Also as in previous studies, *Ifng* gene expression was elevated in NOD mouse LG (Fig. 2A). To further explore its effect on CX3CL1 *in vitro*, we measured *Cx3cl1* gene expression in cultured LGAC from BALB/c mice treated with active recombinant mouse IFN-γ for 2, 4, 8 and 24 hr. *Cx3cl1* gene expression was significantly elevated by 4 hr of IFN-γ treatment and peaked by 8 hr (Fig. 3A). CX3CL1 and CTSS protein levels in LGAC treated with IFN-γ were analysed by quantitation of immunofluorescence (Fig. 3D). CX3CL1 protein in LGAC was elevated at shorter treatment times, peaking at 4 hr and dropping to untreated levels by 24 hr (Fig. 3B). In comparison, CTSS protein was increased by 4 hr of treatment and remained consistently elevated through the treatment period (Fig. 3C). Decreased CX3CL1 in LGAC at 24 hr is consistent with cleavage of the membrane-bound form and release of the soluble form, particularly with the increased CTSS expression measured. In culture medium, a 4-fold increase in CX3CL1 was seen at 24 hr (Fig. 3E). To test whether more CTSS could enhance this recovery, rhCTSS was added to LGAC for 2 hr after pretreatment with IFN-γ for 8 hr to induce higher CX3CL1 levels prior to any observed shedding. CX3CL1 levels in cells, measured by CTCF, were significantly decreased with extracellular CTSS treatment (Fig. 3F), but CX3CL1 levels in the media were unchanged with additional rhCTSS (Fig. 3G).

### IFN-γ treatment increases CX3CL1 in HCE-T cells and culture medium

**Figure 4:**
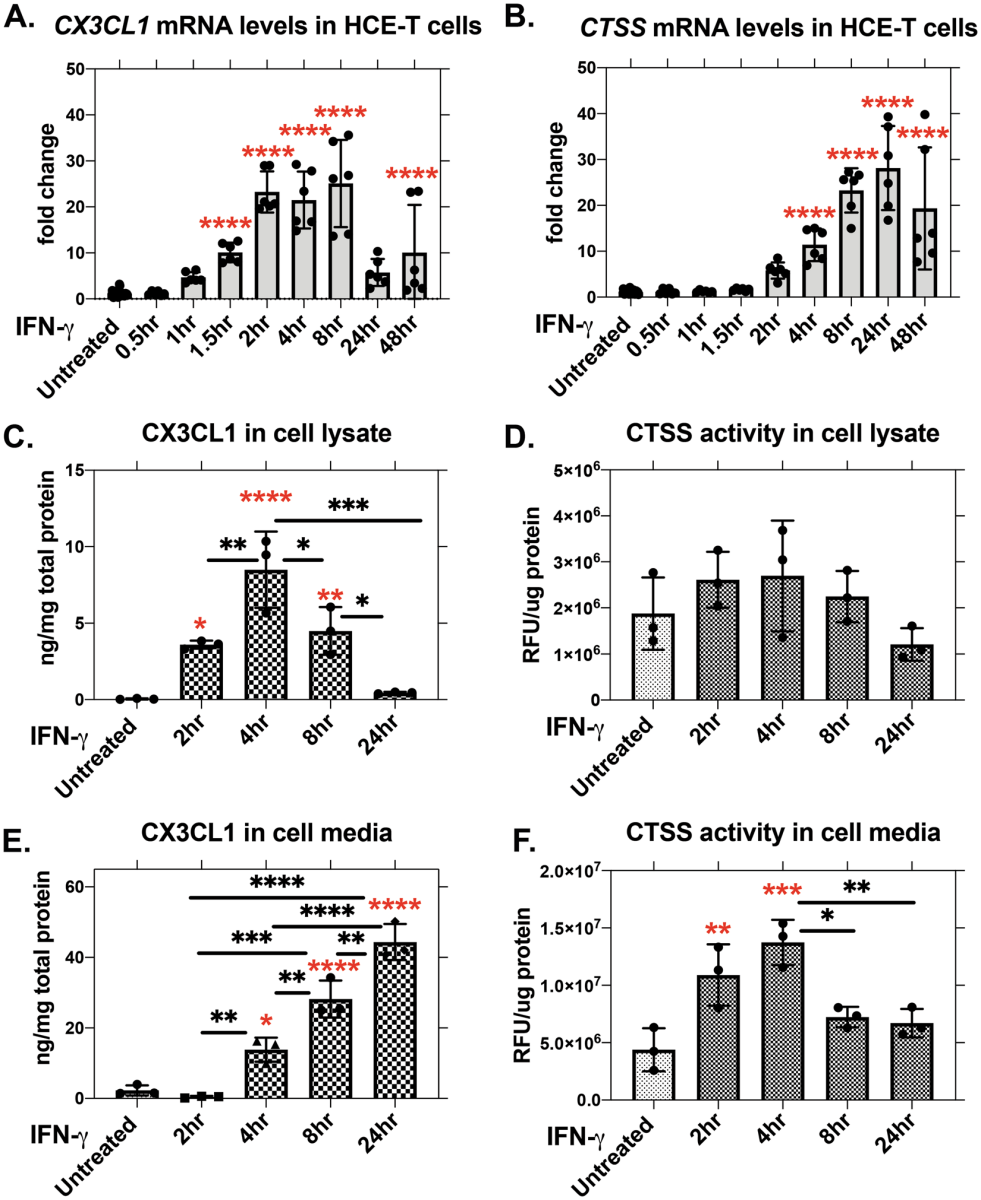
Recombinant human IFN-γ (1 µg/ml) induces both CX3CL1 and CTSS gene and protein expression in HCE-T cells. (**A**) Gene expression of *CX3CL1* was increased by IFN-γ. Relative to untreated: 1.5 hr, p ≤ 0.0001; 2 hr, p ≤ 0.0001; 4 hr, p ≤ 0.0001; 8 hr, p ≤ 0.0001; 48 hr, p ≤ 0.0001. N = 6, (**B**) *CTSS* gene expression was increased by IFN-γ. Relative to untreated: 4 hr, p ≤ 0.0001; 8 hr, p ≤ 0.0001; 48 hr, p ≤ 0.0001. N = 6. (**C**) Soluble CX3CL1 in cell lysate measured by ELISA was increased by IFN-γ. Relative to untreated (red asterisk): 2 hr, p = 0.0256; 4 hr, p ≤ 0.0001; 8 hr, p = 0.0068. Compared between treatments (black asterisk): 2 hr vs. 4 hr, p = 0.0074; 4 hr vs. 8 hr, p = 0.026; 4 hr vs. 24 hr, p = 0.0002; 8 hr vs. 24 hr, p = 0.0244. N = 3. (**D**) No change in CTSS activity in cell lysate was elicited by IFN-γ. N = 3, (**E**) CX3CL1 measured by ELISA in culture medium was increased by IFN-γ (red asterisk): 4 hr, p = 0.0104; 8 hr p ≤ 0.0001; 24 hr, p ≤ 0.0001. Compared between treatments (black asterisk): 2 hr vs. 4 hr, p = 0.0081; 2 hr vs. 8 hr, p ≤ 0.0001; 2 hr vs. 24 hr, p ≤ 0.0001; 4 hr vs. 8 hr, p = 0.0051; 4 hr vs. 24 hr, p ≤ 0.0001; 8 hr vs. 24 hr, p = 0.0022. N = 3. (**F**) CTSS activity in culture medium was increased with IFN-γ treatment. Relative to untreated (red asterisk): 2 hr, p = 0.0049; 4 hr, p = 0.0003. Compared between treatments (black asterisk): 4 hr vs. 8 hr, p = 0.01; 4 hr vs. 24 hr, p = 0.006. N = 3. One-way ANOVA with Dunnett’s multiple comparison was used to compare different times of IFN-γ treatments to untreated, demonstrated with red asterisk. One-way ANOVA with Tukey’s multiple comparison was used to compare between IFN-γ treatments, demonstrated with black asterisk. All data are presented as mean ± SD.

In addition to CTSS, we previously demonstrated increased IFN-γ in tears of male NOD mice^13^. From mouse corneal cross sections, we found that CX3CL1 was mainly distributed in the corneal epithelium. Corrected total cell fluorescence (CTCF) image quantification also revealed a significant accumulation of CX3CL1 in the corneal epithelium of NOD mouse (Supplementary Fig. S1). We analysed whether IFN-γ affected CX3CL1 expression and abundance in human corneal epithelial cells (HCE-T), in parallel with CTSS. HCE-T cells were grown to 70% to 80% confluency, then starved in supplement-free KSFM media for 16–18 hr prior to IFN-γ treatment in complete KSFM media for 0.5, 1, 1.5, 2, 4, 8, 24, and 48 hr. *CX3CL1* gene expression was elevated by 1.5 hr of IFN-γ treatment, peaked at 2 hr and remained elevated through 8 hr of treatment before dropping at 24 hr (Fig. 4A). *CTSS* required longer IFN-γ exposure for increased gene expression, increasing significantly by 4 hr of treatment and remaining elevated through 48 hr (Fig. 4B). To correlate CX3CL1 abundance and CTSS activity in HCE-T medium and lysates with observed changes in gene expression, HCE-T cells were treated with IFN-γ for 2, 4, 8 and 24 hr in complete KSFM medium after starvation. Cell medium and lysates were collected and analysed using ELISA for CX3CL1. CX3CL1 levels in cell lysates were significantly increased at 2 hr of IFN-γ treatment, peaked at 4 hr, then decreased from 8 hr (Fig. 4C), similar to observed patterns of gene expression (Fig. 4A). These effects occurred without a change in CTSS activity in cell lysates (Fig. 4D). However, CX3CL1 in culture medium increased with IFN-γ treatment at 4 hr and continued throughout the 24 hr treatment (Fig. 4E). CTSS activity in the lysates also was significantly elevated at 2 and 4 hr, prior to the accumulation of CX3CL1 in culture medium (Fig. 4F).

### rhCTSS increases CX3CL1 expression in HCE-T cells

**Figure 5:**
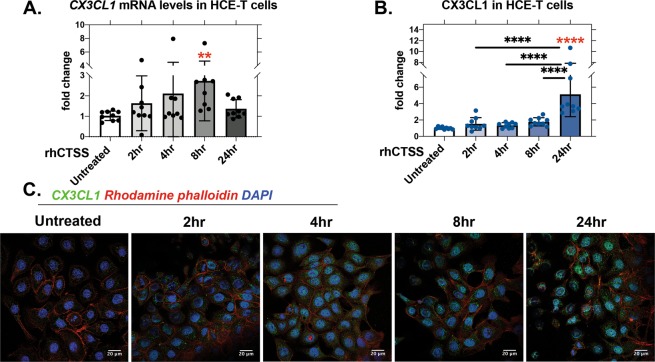
rhCTSS exposure induces CX3CL1 gene and protein expression in HCE-T cells. HCE-T cells grown to 70–80% confluency were starved in supplement-free KSFM basal medium for 16–18 hr and treated with rhCTSS (20,000 RFU/treatment) for 2, 4, 8 and 24 hr. (**A**) *CX3CL1* gene expression was increased by CTSS. Relative to untreated: 8 hr, p = 0.0021 (N = 3), Data points indicate the individual mRNA samples (3 for each N or repeat) presented as points. (**B**) CX3CL1 protein was increased by rhCTSS as determined by quantitation of fluorescence intensity using immunofluorescence labeling and confocal microscopy of images such as those in panel (**C**). Relative to untreated (red asterisk): 24 hr, p ≤ 0.0001. Compared between samples (black asterisk): 2 hr vs. 24 hr, p ≤ 0.0001; 4 hr vs. 24 hr, p ≤ 0.0001, 8 hr vs. 24 hr, p ≤ 0.0001 (N = 3). N = experimental repeats performed on different days, 3 ROIs were analysed under each condition for each repeat and are presented as points. All data are presented as mean ± SD.

CTSS activates the expression and secretion of other cytokines and proteases in HCE-T cells^18^. To evaluate whether CTSS could also induce CX3CL1, HCE-T cells were exposed to recombinant human CTSS (rhCTSS) at activity commensurate with that in SS patient tears, and changes in CX3CL1 gene and protein expression were measured^18^. *CX3CL1* gene expression was significantly increased by 8 hr of rhCTSS, and returned to pre-exposure levels by 24 hr (Fig. 5A). CX3CL1 protein, detected by immunofluorescence and quantified with CTCF method, was significantly increased by 24 hr of rhCTSS (Fig. 5B,C). These results suggest that extracellular CTSS activity can induce CX3CL1 expression in HCE-T cells.

### Extracellular CTSS enhances CX3CL1 shedding in HCE-T cells

**Figure 6:**
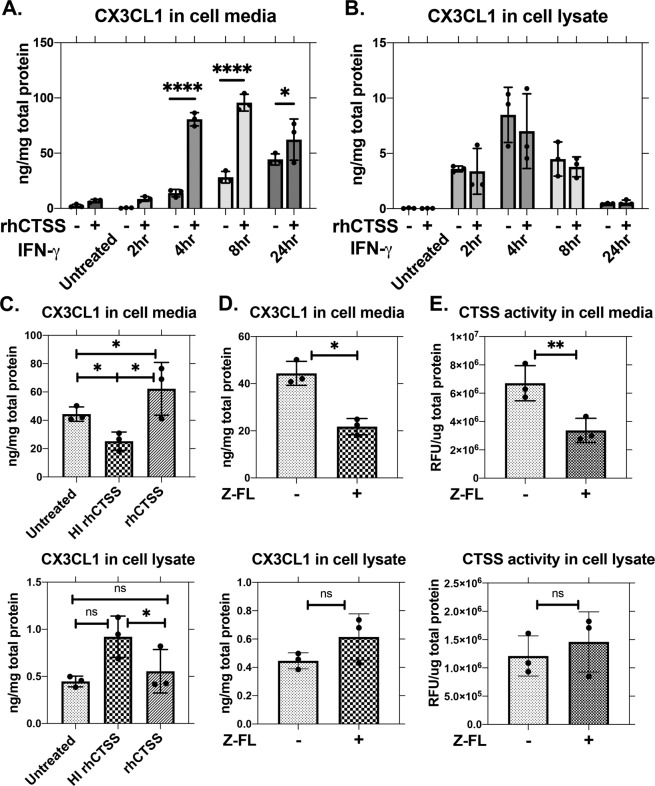
rhCTSS increases CX3CL1 recovery in culture medium. (**A**) HCE-T cells grown to 70–80% confluency and starved in supplement-free KSFM basal medium for 16–18 hr were treated without and with recombinant human IFN-γ (1 µg/ml) for 2, 4, 8 and 24 hr. Additional active rhCTSS (20,000 RFU/treatment) was added during the last 2 hr of IFN-γ treatment. CX3CL1 in culture medium was increased by rhCTSS treatment. Relative to untreated and at the equivalent IFN-γ treatment time: 4 hr: p ≤ 0.0001; 8 hr, p ≤ 0.0001; 24 hr, p = 0.0309, (N = 3). (**B**) CX3CL1 in cell lysates was not significantly affected by rhCTSS (N = 3). (**C**) Heat-inactivated rhCTSS (HI-rhCTSS), at equivalent concentration to active rhCTSS as shown in (A,B) was incubated for 2 hr with HCE-T cells pretreated with IFN-γ for 24 hr. Soluble CX3CL1 in the culture medium was significantly decreased by HI-rhCTSS treatment compared to active CTSS treatment (p = 0.0346) and untreated IFN-γ-induced cells (p = 0.0206). CX3CL1 in cell lysates was significantly increased with HI-rhCTSS treatment compared to CTSS treatment (p = 0.0425). (N = 3). (**D**) HCE-T cells pretreated with IFN-γ for 24 hr were exposed to the CTSS inhibitor, Z-FL (1 µM), for the last 2 hr. Soluble CX3CL1 in the culture medium was significantly decreased with Z-FL treatment (p = 0.0232), but unaffected in cell lysates. (N = 3). (**E**) CTSS activity in culture medium was also significantly reduced by Z-FL treatment, (p = 0.0048) with no changes in CTSS activity in cell lysates. (N = 3) All N values are experimental repeats performed on different days. All data are presented as mean ± SD.

CTSS can generate soluble CX3CL1 though direct proteolysis^38^. To investigate whether active CTSS cleaves membrane-bound CX3CL1 induced in corneal epithelial cells, the abundance of cell-associated and soluble CX3CL1 in HCE-T cell cultures was determined under conditions of differing CTSS activity. To induce high expression of CX3CL1, HCE-T cells were pretreated with recombinant human IFN-γ (1 µg/mL) for 2, 4, 8, and 24 hr (Fig. 4). rhCTSS was then added to IFN-γ treated HCE-T cells for 2 hr. The choice of this relatively short-term exposure of rhCTSS to the cells was intended to minimise the influence of CTSS on gene expression (Fig. 5), and to focus on enzyme-mediated actions of CTSS. Cell medium and cell lysates were collected post-treatment and analysed by ELISA. Soluble CX3CL1 in the cell medium was significantly elevated by rhCTSS, with the highest increases at 4 hr and 8 hr of treatment with IFN-γ (Fig. 6A). Consistent with elevated CX3CL1 in culture medium, CX3CL1 content of cell lysates was slightly decreased by rhCTSS at 4 hr and 8 hr of IFN-γ induction (Fig. 6B). To confirm that effects were due directly to CTSS activity, HCE-T cells exposed to 24 hr IFN-γ were treated with heat-inactivated rhCTSS (HI-rhCTSS) in the last 2 hr of treatment, which decreased CX3CL1 in culture medium relative to rhCTSS or no CTSS. Correspondingly, CX3CL1 in cell lysates was significantly increased with HI-rhCTSS treatment compared to rhCTSS or no CTSS (Fig. 6C). The amount of CX3CL1 present in culture medium and cell lysates of HCE-T cells exposed to IFN-γ for 24 hr and then treated without or with Z-FL, a specific CTSS inhibitor, in the final 2 hr also showed that the CX3CL1 content of the culture medium was significantly reduced by Z-FL treatment while CX3CL1 in cell lysates was not significantly affected (Fig. 6D). At the same time, CTSS activity in the cell culture medium was decreased significantly with Z-FL, with no change in CTSS in cell lysates (Fig. 6E**)**.

### *Cx3cr1* expression and CX3CR1+ immune cells are increased in male NOD LG

**Figure 7:**
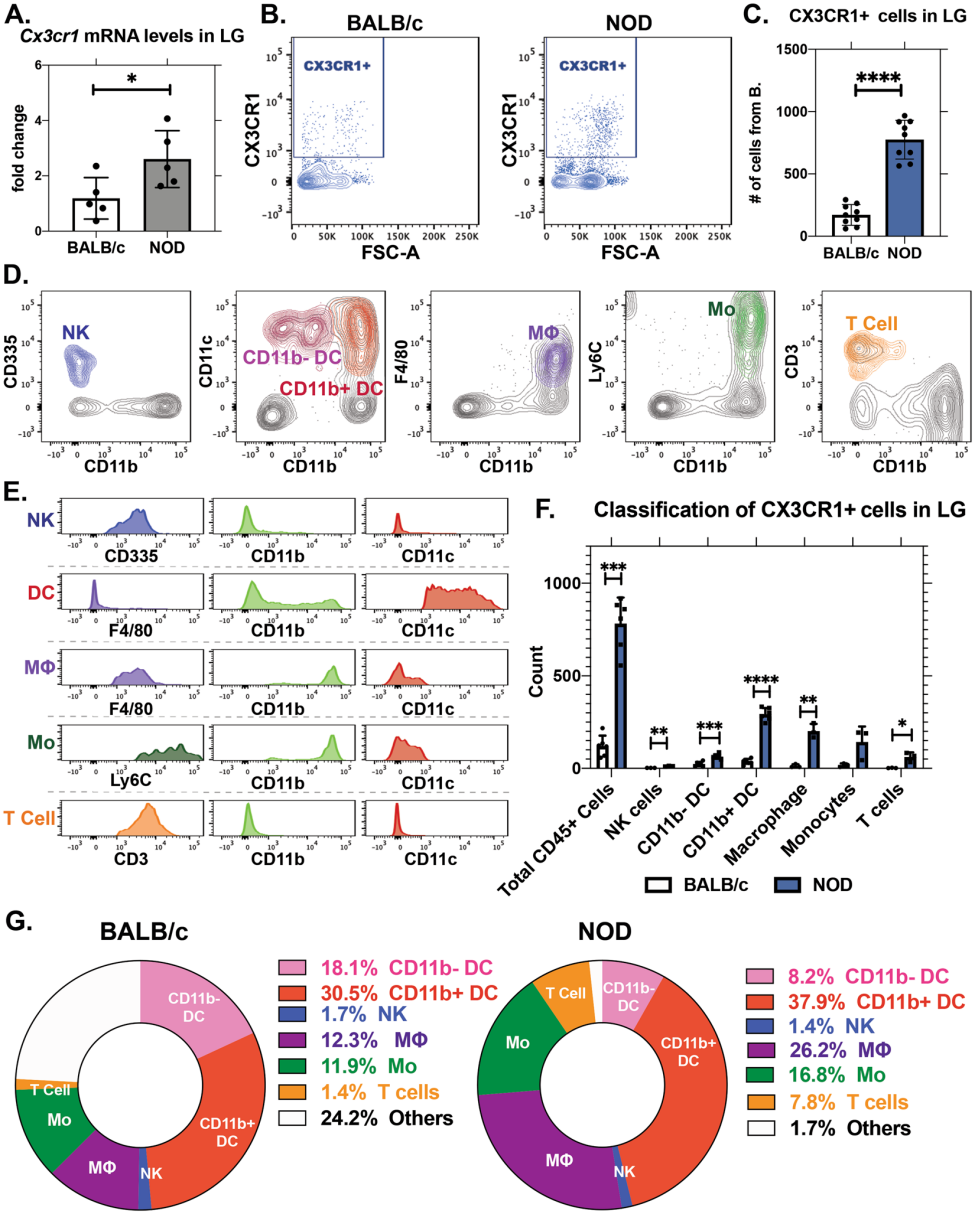
CX3CR1+ immune cells are increased in NOD mouse LG. Flow cytometry was performed on isolated cells from LG from BALB/c and NOD mice. (**A**) *Cx3cr1* gene expression in LG was significantly increased in NOD mice relative to BALB/c mice. p = 0.0375, N = 5 mice. (**B**) CX3CR1+ cells from BALB/c and NOD mouse LG after gating for live CD45+ singlets. (**C**) Absolute cell numbers of CX3CR1+ cells from panel (B) were increased in NOD mouse LG compared to BALB/c. p ≤ 0.0001, N = 9 mice. (**D**) Validation of immune cell identities in mouse LG. Populations are demonstrated in NOD mouse LG, after gating for live singlet CD45+. NK = natural killer cells, DC = dendritic cells, MΦ = macrophages, Mo = monocytes. (**E**) Histograms from the respective gated populations, supporting immune cell population validation. (**F**) The CX3CR1+ CD45+ cell population was further identified based on the gating and validation method illustrated in panel (D,E). Absolute numbers of NK cells (p = 0.0015), CD11b− (p = 0.0002) and CD11b+ dendritic cells (p ≤ 0.0001), macrophages (p = 0.0012) and T cells (p = 0.0159) were significantly elevated in NOD mice. N = 3~6, mice per immune cell type. (**G**) CX3CR1+ cell cells in NOD mouse LG are altered compared to BALB/c mouse LG. All data are presented as mean ± SD.

CX3CL1 contributes to immune cell recruitment through interactions with its receptor, CX3CR1, which is largely expressed on immune cells. We analysed CX3CR1 expression and enrichment on immune cells in NOD and BALB/c LG. In whole LG, *Cx3cr1* gene expression was significantly elevated in NOD mice compared to BALB/c (Fig. 7A). To explore whether increased *CX3CR1* gene expression was related to increased numbers of CX3CR1+ cells, LGs were isolated and processed as single cell suspensions for flow cytometry profiling. The immune cell (CD45+) population expressing high CX3CR1 protein is shown in the gated area in Fig. 7B and quantified in Fig. 7C. A significantly greater number of CX3CR1+ immune cells were seen in NOD mouse LG relative to BALB/c (Fig. 7C) within same number of events. To further identify the CX3CR1+ populations, we evaluated the presence of NK cells (CD335+ CD11b− CD11c−), dendritic cells (CD11c+ F4/80-), macrophages (CD11b+ F4/80+ CD11c-), monocytes (CD11b+ Ly6C+ CD11c−) and T-cells (CD3+ CD11b− CD11c−). Figure 7D shows the gated populations in colour overlaying the total immune cell population in grey. The gating strategy is outlined in Supplementary Fig. S3. The histograms in Fig. 7E validated the identity of each respective gated population by demonstrating related marker expression. T-cells were identified by high CD3+ levels, dendritic cells by CD11c, natural killer cells by CD335, macrophages by F4/80 and monocytes by Ly6C. With this strategy, we identified the frequencies and percentages of cells in each CX3CR1+ population, and determined the differences in these cell types between BALB/c and NOD mouse LG. The absolute numbers of CX3CR1+ NK cells, CD11b+ and CD11b− dendritic cells, macrophages and T-cells were all significantly elevated in NOD mice LG (Fig. 7F). Among CX3CR1+ immune cell populations in NOD LG, the percentage of T cells increased by 5-fold and macrophages by 2-fold (Fig. 7G). These results suggest a population of CX3CR1+ immune cells showing increased migration into LG tissue in autoimmune dacryoadenitis, concurrent with CX3CL1 and CTSS elevation in diseased LG and tears.

## Discussion

**Figure 8:**
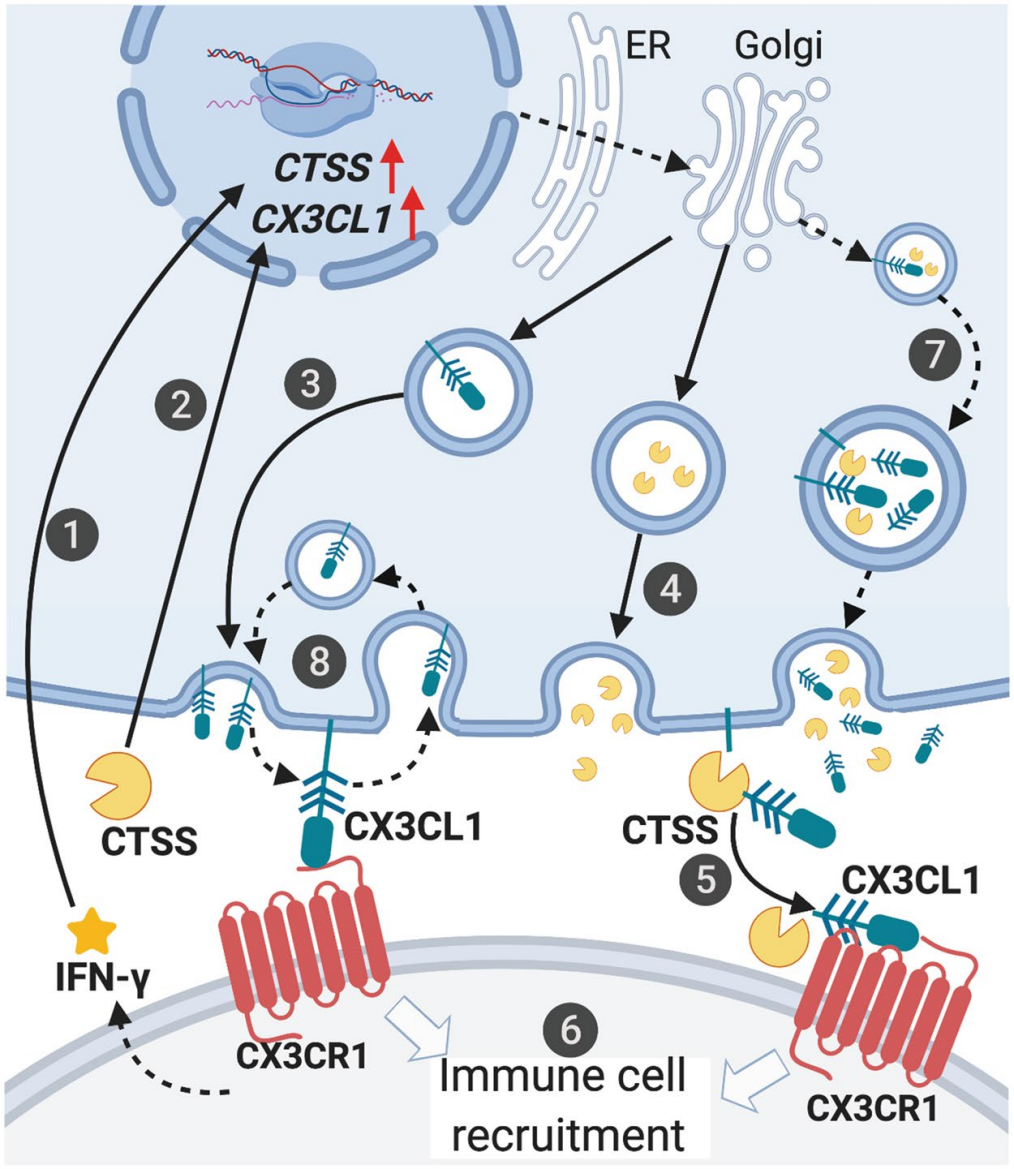
Proposed mechanisms implicated in CX3CL1 induction, cleavage and recruitment of CX3CR1+ immune cells in autoimmune dacryoadenitis. 1. Pro-inflammatory cytokines including IFN-γ induce gene and protein expression of *CX3CL1* and *CTSS*. 2. CTSS activity also contributes to upregulation of *CX3CL1* expression. 3. Induced CX3CL1 is synthesised as the membrane-bound form and trafficked to the plasma membrane where it is exposed. 4. Upregulated CTSS is secreted, leading to an accumulation of active CTSS in areas rich in CX3CL1. 5. Active CTSS enhances shedding of membrane-bound CX3CL1 and generates soluble CX3CL1. 6. Soluble and membrane-bound CX3CL1 interact with CX3CR1 to recruit immune cells to sites of inflammation. 7. Intravesicular cleavage of membrane-bound CX3CL1 by increased CTSS present in both basolaterally-targeted and apically-targeted vesicles may also occur, increasing CX3CL1 shedding into the interstitium and tears. 8. Restrained constitutive recycling of CX3CL1 by alterations in actin cytoskeleton may increase membrane-bound CX3CL1 on the plasma membrane, also promoting shedding of CX3CL1. Solid arrows highlight mechanisms suggested as primary explanations in the study while dashed arrows are alternative mechanisms. Figure created with BioRender.

In NOD mice, autoimmune dacryoadenitis associated with elevated CTSS activity in LG and tears is correlated with increased CX3CL1 in LG, cornea and tears, and with increased CX3CR1+ immune cells in LG. Exposure of cultured LGAC to IFN-γ coordinately induces expression of CX3CL1 and CTSS. Moreover, rhCTSS at activities found in SS patient tears increases CX3CL1 expression in HCE-T cells and recovery of CX3CL1 in culture medium. Figure 8 shows the proposed relationships between IFN-γ, CTSS, CX3CL1 and CX3CR1, highlighting mechanisms that may occur both within LG and on the ocular surface.

The elevated CX3CL1 found in the tears of NOD mice is likely due in part to the presence of CX3CL1 in apical secretory compartments also enriched in CTSS (Fig. 2B), which may expose membrane-bound CX3CL1 to increased active CTSS also present in these compartments for shedding in tears^13^. IFN-γ is also elevated in tears of NOD mice with autoimmune dacryoadenitis and in patients with primary SS^13^. The demonstration that both IFN-γ and CTSS exposure to HCE-T cells increase recovery of CX3CL1 in culture medium is consistent with a model where these tear constituents continue to elicit inflammatory responses in cornea and other ocular surface epithelia, which in turn can further enhance shedding of CX3CL1 into tears.

Our finding is the first to report CX3CL1 in tears in a model of autoimmune dacryoadenitis. CX3CL1 is elevated in tears of general dry eye^39^ and evaporative dry eye^40^ patients, but SS patient tears have not been evaluated. CX3CL1 in tears could participate in ocular surface pathology in SS by recruiting immune cells to the ocular surface^41,42^ and possibly interfering with corneal nerves. CX3CL1 has been previously implicated in development of autoimmune exocrinopathy in the LG in another murine model^31^, but its function and mode of generation were not addressed.

We propose that the accumulation of CX3CR1+ immune cells in NOD mouse LG is driven by increased shedding of CX3CL1 from LGAC, an effect driven in part by elevated CTSS present in the interstitium of the LG. CX3CL1 is clearly increased in basolateral membranes and basolateral vesicles in LGAC from NOD mice (Fig. 2B). While increased LGAC CTSS is clearly secreted apically^8,10^, its cellular overexpression is expected to increase its abundance in tissue interstitium through release at basolateral membranes. CTSS is normally present in all cell lysosomes and in late endosomes and lysosomes of professional antigen-presenting cells. This localisation is associated with its mannose-6 phosphorylation (M6P), resulting in its capture by M6P-receptors and active sorting to endolysosomal compartments. Overexpression of cathepsins results in increased amounts of cathepsins lacking M6P tags, since this modification is rate limiting^43^. These unmodified cathepsins can be secreted by bulk flow mechanisms to the extracellular space where they participate in degradation of extracellular matrix and other inflammatory functions^17,43^. CTSS is one of the only cathepsins that is equally active at a neutral (extracellular) pH, as well as in the acidic environment of the lysosomes^17^. Elevated LG CTSS associated with increased IFN-γ or other pro-inflammatory cytokines may therefore yield additional extracellular CTSS in proximity to overexpressed CX3CL1 that is enriched in basolateral vesicles and membranes. Figure 8 summarises this aspect of our model.

IFN-γ is a known inducer of CTSS, and is elevated in tears and LG of diseased NOD mice^13^. IFN-γ exposure *in vitro* also modestly increases CTSS in HCE-T cells and primary LGAC^13^. The work herein has reconfirmed these findings, further showing that IFN-γ induction increases CX3CL1 gene and protein expression. The time course for detection of increased CX3CL1 expression relative to CTSS expression in LGAC (Fig. 3) and HCE-T cells (Fig. 4) shows that *CX3CL1* gene expression precedes *CTSS* (Fig. 4). However, increased recovery of CX3CL1 in culture medium lags relative to increased CTSS activity (Fig. 4). CTSS activity itself increases CX3CL1 in HCE-T cells, although these effects are not detectable until later times (Fig. 5). The initial increase in CX3CL1 expression appears related to IFN-γ exposure, rather than to the increased expression of CTSS. The burst of CTSS activity detected in the culture medium of HCE-T cells that precedes the increased CX3CL1 recovery in culture medium may reflect secretion of existing cellular stores of CTSS prior to the induction of additional CTSS expression to replenish intracellular stores^44^. In this study, we focused mainly on IFN-γ, although SS cytokines including TNF-α and IL-1β may also affect CX3CL1.

We did not focus extensively on the role of CTSS in LGAC shedding of CX3CL1 in cultured LGAC beyond initial findings in Fig. 3. This choice was due in part to the large number of mice required for cultured LGAC preparations, and in part to the lower extent of CX3CL1 protein expression elicited in LGAC by IFN-γ (1.9-fold) relative to HCE-T cells (196-fold). However, several findings in HCE-T cells suggest that CTSS directly cleaves CX3CL1, including observations that added CTSS increased CX3CL1 in culture medium, while CX3CL1 was decreased in culture medium by the CTSS inhibitor, Z-FL (Fig. 6). Moreover, heat-inactivated rhCTSS did not elicit CX3CL1 release into culture medium (Fig. 6). Other proteases upregulated in SS such as MMP-9 may also contribute to increased tissue and tear CX3CL1. MMP-9 is also upregulated by exposure of HCE-T cells to rhCTSS activity but this effect requires 24 hr of treatment^18^. We used only 2 hr of rhCTSS treatment of HCE-T cells to minimise later effects of CTSS on other signalling pathways.

An alternative explanation for the increased CX3CL1 released into tears and possibly interstitium beyond active cleavage by elevated CTSS is through altered intracellular trafficking. Membrane-bound CX3CL1 is rapidly recycled between the plasma membrane and endocytic compartments, regulating its availability for processing^45^. Constitutive endocytosis of CX3CL1 is reported to protect membrane-bound CX3CL1 from cleavage by metalloproteases^46^. When actin cytoskeleton is disrupted, more interaction between CX3CL1 and metalloproteases occurs, resulting in shedding of soluble CX3CL1^47^. The actin cytoskeleton is intimately associated with exocytosis and endocytosis in LGAC. We have reported multiple changes in LGAC secretory pathways and actin filament organisation in NOD mouse^9,48^. Such a mechanism might account for increased CX3CL1 in tears, and the increased CX3CL1 in interstitium implied by detection of increased CX3CL1 at the basolateral membranes of NOD mouse LGAC and the increased accumulation of CX3CLR+ immune cells in NOD mouse LG. Future studies utilising selective protease inhibitors *in vivo* may elucidate the role of these different processes in regulation of the CX3CL1-CX3CR1 axis in LG.

Several CTSS inhibitors are in clinical trials for autoimmune diseases, including RWJ-445380 (www.clinicaltrials.gov, Identifier: NCT00425321) and RO5459072 (www.clinicaltrials.gov, Identifier: NCT02701985). These therapeutic studies have focused primarily on assessment of MHC II-related endpoints such as Iip10 accumulation or *H2-*Ab1 gene expression as indicators of therapeutic efficacy^49^. CX3CL1 levels and CX3CL1+ immune cell counts in tissues of interest may serve as additional endpoints for CTSS activity. The CX3CL1-CX3CR1 axis is implicated in RA pathogenesis, where CX3CL1 is expressed in fibroblast-like synoviocytes and endothelial cells in RA synovium. Accumulation of CX3CR1+ T-cells and macrophages in RA synovial tissue is also associated with upregulation of CX3CL1^50,51^. Since comparable changes are observed in the LG of NOD mice, the CX3CL1-CX3CR1 axis may participate similarly in autoimmune dacryoadenitis. CX3CL1-CX3CR1 also participates in immune cell homing to the conjunctiva during ocular inflammation induced by benzalkonium chloride^42^. Currently, an anti-CX3CL1 monoclonal antibody therapy, KANAb001 (E6011), is undergoing phase I and II clinical trial in Japan for RA and Crohn’s disease^52^. CX3CL1 may therefore represent another potential therapeutic target in SS to mitigate autoimmune exocrinopathy.

In conclusion, CX3CL1 is elevated in tears, corneas and LG of a murine model recapitulating the ocular symptoms of SS, and this elevation is correlated with increased CTSS and IFN-γ. CTSS-mediated induction/cleavage of CX3CL1 may contribute to ocular surface and LG inflammation in SS. These findings suggest CX3CL1 as a predictive outcome biomarker for CTSS and IFN-γ targeted SS therapies. Given its role in immune cell homing by recruitment of CX3CR1+ immune cells, CX3CL1 may also serve directly as a therapeutic target.

## Methods

### Reagents

Carbachol (CCh) (CAS No. 51832) was from Sigma-Aldrich (St. Louis, MO). The cathepsin S activity kit (#K144), rabbit polyclonal cathepsin S antibody (#6686) and active recombinant human cathepsin S protein (rhCTSS, #7526) were from Biovision Inc. (Milpitas, CA). RNA extraction kits (#73404, #74134) were from Qiagen (Hilden, Germany). Rabbit polyclonal CX3CL1 antibody (#ab25088) was from Abcam (Cambridge, MA). Rhodamine phalloidin, donkey anti-rabbit Alexa Fluor 488 secondary antibody and ProLong gold antifade mountant were from Invitrogen (Grand Island, NY). DAPI was from Molecular Probes, Inc. (Eugene, OR). Keratinocyte serum-free medium (KSFM) supplemented with human recombinant epidermal growth factor and bovine pituitary extract (#17005042) were from Life Technologies (Grand Island, NY). Human (#DCX310) and mouse (#MCX310) CX3CL1/fractalkine Quantikine ELISA kits were from R&D systems (Minneapolis, MN). Reverse transcriptase and primers including human CTSS (Hs00175407_m1), CX3CL1 (Hs00171086_m1), GAPDH (Hs02786624_g1); mouse Ctss (Mm01255859_m1), Cx3cl1 (Mm00436454_m1), Cx3cr1 (Mm02620111_s1), Ifng (Mm00801778_m1), Tnf (Mm00443258_m1), Gapdh (Mm99999915_g1) and TaqMan universal PCR master mix (#4440040) were from Applied Biosystems (Grand Island, NY). Recombinant mouse IFN-γ (#IF005), human IFN-γ (#IF002) and Amicon ultra centrifugal filters (#ufc501096) were from EMD Millipore (Burlington, MA). Z-FL-COCHO (Z-FL, #A13502) was from Adooq Bioscience (Irvine, CA). Bio-Rad protein assay dye was from Bio-Rad (#5000006, Hercules, CA). BCA reagents (#23235) and Live/Dead fixable aqua dead cell stain (#L34966) were from Thermo Fisher Scientific (Rockland, IL). Bovine serum albumin (#2905) was from Calbiochem (Billerica, MA). TruStain FcX Plus (#156604) and the following fluorescently-conjugated antibodies for flow cytometry (anti-CD45, #103127; anti-CD11b, #101205; anti-CD11c, #117317; anti-CD335, #137603, anti-CX3CR1, #149007; anti-F4/80, #123110; anti-Ly6C, #128007) were from Biolegend (San Diego, CA).

### Mice

BALB/cJ (000651) and NOD ShiLtJ (001976) mice were from Jackson Laboratories (Sacramento, CA). All mouse work utilised male mice aged 16–17 weeks. Mouse age and sex were carefully selected based on the ability to develop the autoimmune dacryoadenitis and reduced tear flow characteristic of SS with a low diabetic risk. Lymphocytic infiltration in the LG is significantly correlated with disease development^8^. Male NOD mice consistently develop ~20% infiltration of the LG by 16 weeks^8^, while female NOD mouse are reported in one study to develop less than 1% infiltration of the LG by 24 weeks^53^. Our analysis of LG disease in female NOD mice aged 2–7 months by LG histology (Supplementary Fig. S4) and using gene expression of inflammatory indicators in LG (Supplementary Fig. S5), comparable to this previous characterization in male NOD mice over a comparable time period^8^, showed no indications of disease in the LG. As NOD mice develop diabetes in an age- and sex-dependent manner, a comprehensive evaluation of age-related blood-glucose levels in a previous cohort of male NOD mice also showed that no mice evaluated between 1 to 6 months of age had non-diabetic blood-glucose levels (below 250 mg/dL^54^) (Supplementary Fig. S6). Female NOD mice are reported to have an earlier onset of diabetes at 10 to 12 weeks, compared to males, which begin at approximately 20 weeks of age^55^. A higher cumulative incidence of diabetes was observed in 30 week females (70–80%), comparing to males of the same age (20%)^55–57^. In 16 week old NOD mice, females have a 20% diabetic rate, while less than 5% males develop diabetes^58^. Thus, male NOD mice are preferred for evaluation of ocular symptomatology of SS without complications. All animal procedures were in accordance with the Guiding Principles for the Care and Use of Laboratory Animals (8^th^ edition) and approved by USC’s Institutional Animal Care and Use Committee.

### Tear collection

As described^9^, mouse LGs were surgically-exposed and stimulated topically with carbachol (CCh, 3 µL, 50 µM). 2 µL microcaps pipettes were placed at the lateral canthus of the eyes for tear collection. Each gland was stimulated 3 times, with 5 min collection each.

### Mouse primary cell culture and treatments

As described^13^, LG isolated from 10 male BALB/c mice were pooled, washed with Ham’s medium, and minced into 1 mm^3^ pieces. Pieces were transferred into 150 mL Erlenmeyer flasks and incubated with shaking in H-E medium and CHD medium at 37 °C. Supernatants were collected after each incubation. Cells were pelleted by centrifugation and filtered through 100 μm cell strainers. Filtrate was layered on 5% Ficoll and centrifuged at 300 × g for 10 min. Acinar cells were pelleted, washed with Ham’s medium and resuspended in Matrigel-supplemented Peter’s serum-free culture medium^9^. Cells were seeded at 2 × 10^6^ cells per well in 12-well plates and coverslip-coated 24-well plates. Cultured mouse LGACs were treated with 200 U/ml recombinant mouse IFN-γ after 2 hr of seeding.

### HCE-T cell culture and treatments

The SV-40 transformed human corneal epithelial cell line, HCE-T cells, was obtained from the RIKEN Cell Bank, Japan (RCB2280) and cultured in complete KSFM supplemented with human recombinant EGF, bovine pituitary extract and gentamycin. Cells were starved for 16–18 hr in supplement-free KSFM prior to treatments. Recombinant human IFN-γ (1 µg/mL) treatment was in complete KSFM media.

### CTSS treatment

The activity of recombinant human CTSS (rhCTSS) used for treatments was equal to the enzymatic activity level detected in the 90–95^th^ percentile in SS patient tears^10^. CTSS activity was tested prior to each treatment and maintained at 20,000 RFU per treatment.

### Analysis of gene expression in cells and tissues

RNA from mouse primary LGACs and HCE-T cells was extracted using the RNeasy Plus Mini Kit. RNA from the corneal button and LG were extracted using RNeasy Plus Universal Mini Kit. The reverse transcription reaction used the reverse transcription kit, with 2 μg total RNA from corneal buttons, mouse primary LGACs and HCE-T cells, and 4 μg total RNA from LG for each 50 μL reaction. cDNA was obtained using GeneAmp PCR System 9700 with incubation cycles of 25 °C (10 min), 48 °C (30 min) and 95 °C (5 min). Real-time qPCR was carried out with the QuantStudio 12K Flex Real-Time PCR System with GAPDH as an internal control. Human and mouse primers to CX3CL1, CX3CR1, CTSS, IFN-γ and TNF-α were used. Reaction conditions and calculation methods were as previously described^13^.

### Tear CTSS activity measurements

For each assay, tears collected from two mice were pooled and diluted. Each sample was split in half to assay both CTSS activity and CX3CL1 content (below). Activity was measured with the CTSS activity fluorometric assay kit according to the manufacturer’s instructions^7^. Fluorescence was measured using 400/505 nm excitation/emission filters in a SpectraMax iD3 (Molecular Devices, San Jose, CA). Protein concentration was measured using the Bio-Rad protein assay.

### ELISA measurements of CX3CL1

For measurement of tear CX3CL1, tears collected from two mice were pooled and diluted as above. For measurement of serum CX3CL1, blood was collected from mice via cardiac puncture and centrifuged at 1000 × g, 10 min, 4 °C for isolation of serum. For CX3CL1 measurements in tissue, mouse corneal buttons and LG were collected, rinsed with PBS and homogenised in PBS with a BeadBlaster 24 microtube homogeniser (Benchmark Scientific, Edison, NJ) at 7 M/S speed for 1 min for 2 cycles at 30 s intervals. Tissue lysates were collected and centrifuged at 5000 × g, 5 min, 4 °C. For measurement of CX3CL1 in LGAC culture medium, medium was collected and centrifuged at 1000 × g, 10 min, 4 °C. For measurement of CX3CL1 in HCE-T cell culture medium, medium was collected and centrifuged at 500 × g, 10 min, 4 °C. Both clarified supernatants were concentrated with protein centrifugal filters. For measurement of CX3CL1 in HCE-T cells, cells were collected by scraping, pelleted and lysed in cell lysis buffer for 30 min on ice with constant vortexing. Samples were spun down at 5000 × g, 5 min, 4 °C. Supernatants were concentrated with centrifugal filters. CX3CL1 levels in all samples were measured using either the mouse or human CX3CL1/fractalkine Quantikine kit as appropriate and according to the manufacturer’s protocol. Plates were read using the SpectraMax iD3. Biorad or BCA assays were used for total protein in each sample.

### Cell and tissue processing and confocal fluorescence microscopy

Cells and LG were processed for immunofluorescence labeling as described previously^13^. Images were acquired with a Zeiss LSM 800 equipped with an Airyscan detector. Corrected total cell fluorescence (CTCF) Image quantification^37,59^ was performed with an open source image processing pipeline^60^ using python and ImageJ. Detailed methods are in Supplementary Information.

### Flow cytometry

LG from individual mice were isolated and digested in multiple rounds of incubation in H-E medium and CHD medium. Supernatants were collected and passed through a 70 µm cell strainer. Cells were pelleted and resuspended in cell-staining media at a concentration of 5 to 10 × 10^6^ cells/mL. Cells were treated with mouse TruStain FcX Plus (1 µg per 10^6^ cells) for 10 min to block Fc-receptors. Live/Death fixable aqua dead cell stain was added to the cell suspension (1 µL per 10^6^ cells). Fluorescently-conjugated antibodies including anti-CD45, anti-CD11b, anti-CD11c, anti-CD335, anti-CX3CR1, anti-F4/80 and anti-Ly6C were added to the cell suspension at optimised concentrations and incubated on ice in the dark for 30 min. Cells were washed twice, centrifuged at 350 × g for 5 min, and resuspended in cell-staining buffer for flow analysis on a BD LSR Fortessa X20 (Franklin Lakes, NJ). Unstained live cells were used as controls to identify positive staining.

### Statistics

All statistical analyses were performed using GraphPad Prism 8.3.0 software (San Diego, CA). Data normality was assessed by Kolmogorov-Smirnov, D’Agostino and Person omnibus, and Shapiro-Wilk normality tests. A two-tailed unpaired Student’s t-test was used to compare BALB/c and NOD mouse data sets. Two-tailed paired t-tests and two-tailed ratio paired t-tests were used to compare between sets for analysis of gene expression and ELISA in cultured cells. A one-way ANOVA with Dunnett’s comparison was used to compare multiple timepoints to the control group. A one-way ANOVA with Tukey’s multiple comparison was used to compare effects between multiple groups with different treatment times. A two-way ANOVA with Sidak’s multiple comparison was used to compare untreated and CTSS-treated HCE-T cell groups during different conditions of IFN-γ exposure. A Kruskal-Wallis test was used for multiple comparisons on non-normal distributed data. The criterion for statistical significance was set at p ≤ 0.05. In all graphs, ns, p > 0.05; *p ≤ 0.05; **p ≤ 0.01; ***p ≤ 0.001; ****p ≤ 0.0001.

## Supplementary information

Supplementary information.
