# Supplementary material for: Cathepsin S activation contributes to elevated CX3CL1 (fractalkine) levels in tears of a Sjögren’s syndrome murine model
Source: Sci Rep. 2020 Jan 29;10:1455. doi: 10.1038/s41598-020-58337-4 (PMC6989636; doi:10.1038/s41598-020-58337-4)
Supplement: Supplementary file 1 — Supplementary information. [file 41598_2020_58337_MOESM1_ESM.pdf]

Supplementary Information

Cathepsin S activation contributes to elevated CX3CL1 (fractalkine) levels in tears of a Sjögren's syndrome murine model

Runzhong Fu[,2], Hao Guo[1,2], Srikanth Janga[2], Minchang Choi[1,2], Wannita Klinngam[1,2], Maria C. Edman[2] and Sarah F. Hamm-Alvarez[1,2*]

[1]Department of Pharmacology and Pharmaceutical Sciences, School of Pharmacy and [2]Department of Ophthalmology, Roski Eye Institute, Keck School of Medicine, University of Southern California, Los Angeles, CA, 90033, USA

*Address Correspondence to: shalvar@usc.edu

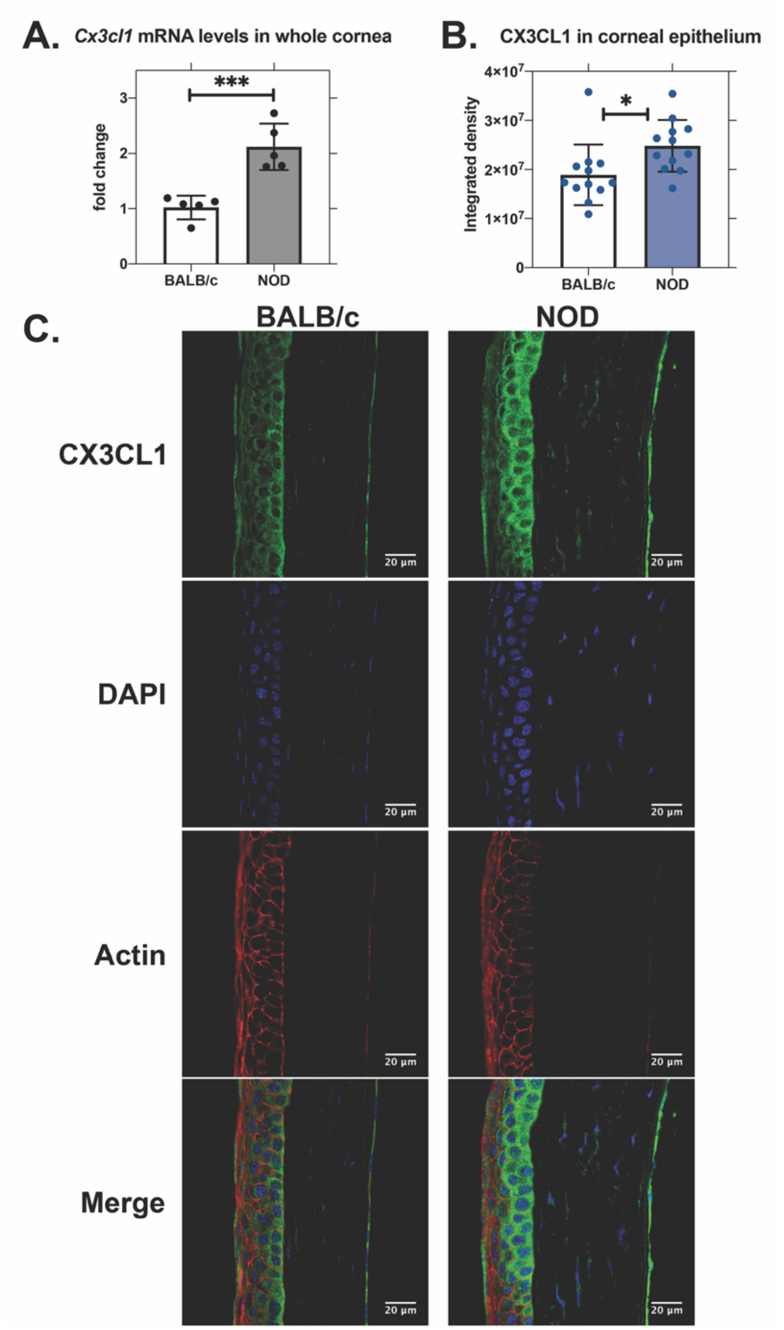

Supplementary Fig. S1. *Cx3cl1* is elevated in the corneal button of NOD mouse. A. *Cx3cl1* gene expression was significantly elevated in the corneal buttons of NOD mice relative to BALB/c mice (p=0.0008, N=5). N, mRNA sample pooled from 2 mouse. **B.** CX3CL1 immunofluorescence in corneal epithelium, quantified from **C**, showed a significant increase in NOD mice (p=0.0195, N=4). N, mouse number. 3 ROIs per sample were quantified, presented as points on the graph. **C.** Confocal fluorescence microscopy shows CX3CL1 protein distribution in the plasma membrane and membrane organelles in the corneal epithelium. Green, CX3CL1; red, rhodamine phalloidin labeling F-actin; blue, DAPI. All data are presented as mean ± SD.

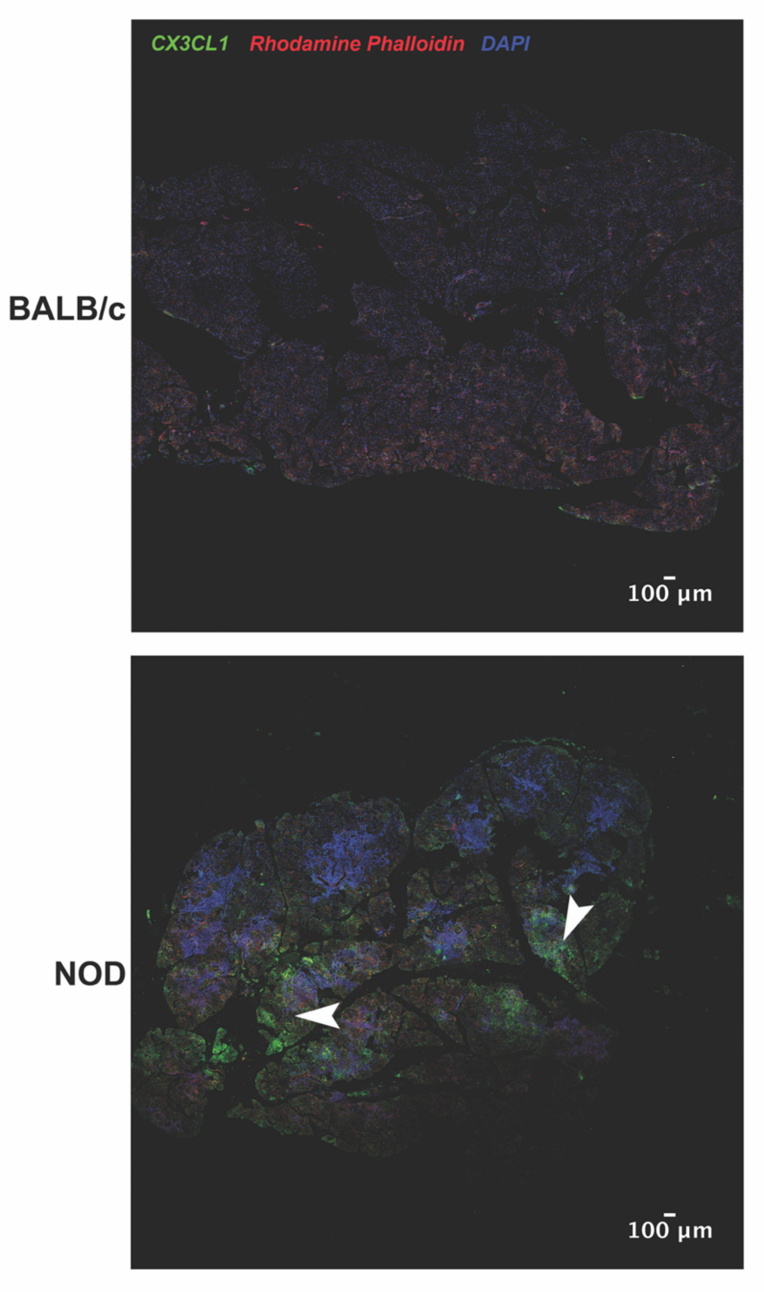

Supplementary Fig. S2. CX3CL1 is increased in a cross section of NOD mouse LG compared to BALB/c mouse LG. Arrows indicate sites of increased CX3CL1 (green).

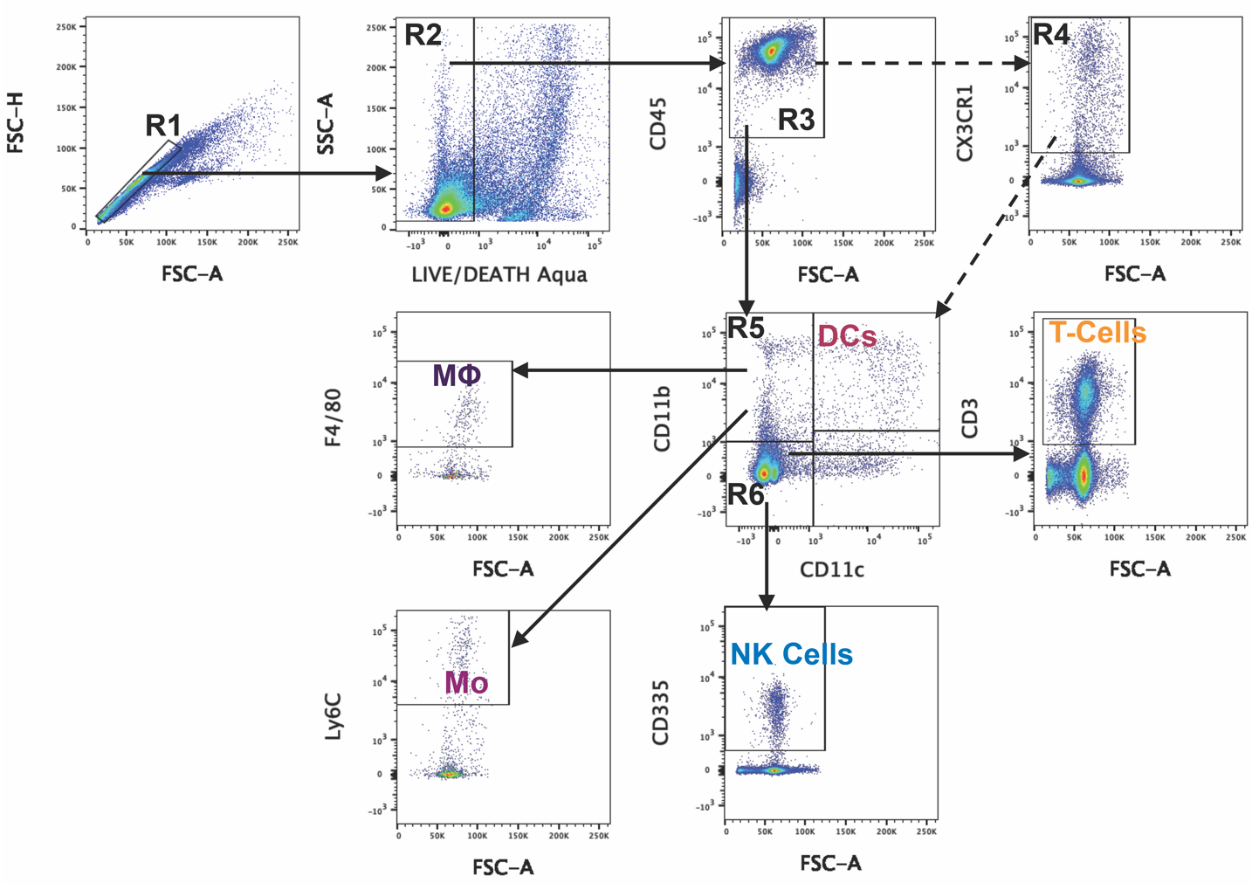

Supplementary Fig. S3. Gating strategy to identify immune cell populations. As reported[1], the dot plot shows selected windows and the gating strategy applied to identify immune cell populations in the LG, shown with solid arrows. The gating strategy begins on the top row, **R1** to **R3** gate for single live immune cells, which were then gated with CD11c and CD11b to identify CD11c+ dendritic cells. CD11b+ CD11c- cells in **R5** were further identified as macrophages (MΦ) and monocytes (Mo) with F4/80 and Ly6C. CD11b- CD11c- cells in **R6** were distinguished as T-cells and nature killer (NK) cells by CD3 and CD335 markers, respectively. Broken arrows show additional gating steps to identify immune cell population within CX3CR1+ cells in **R4.**

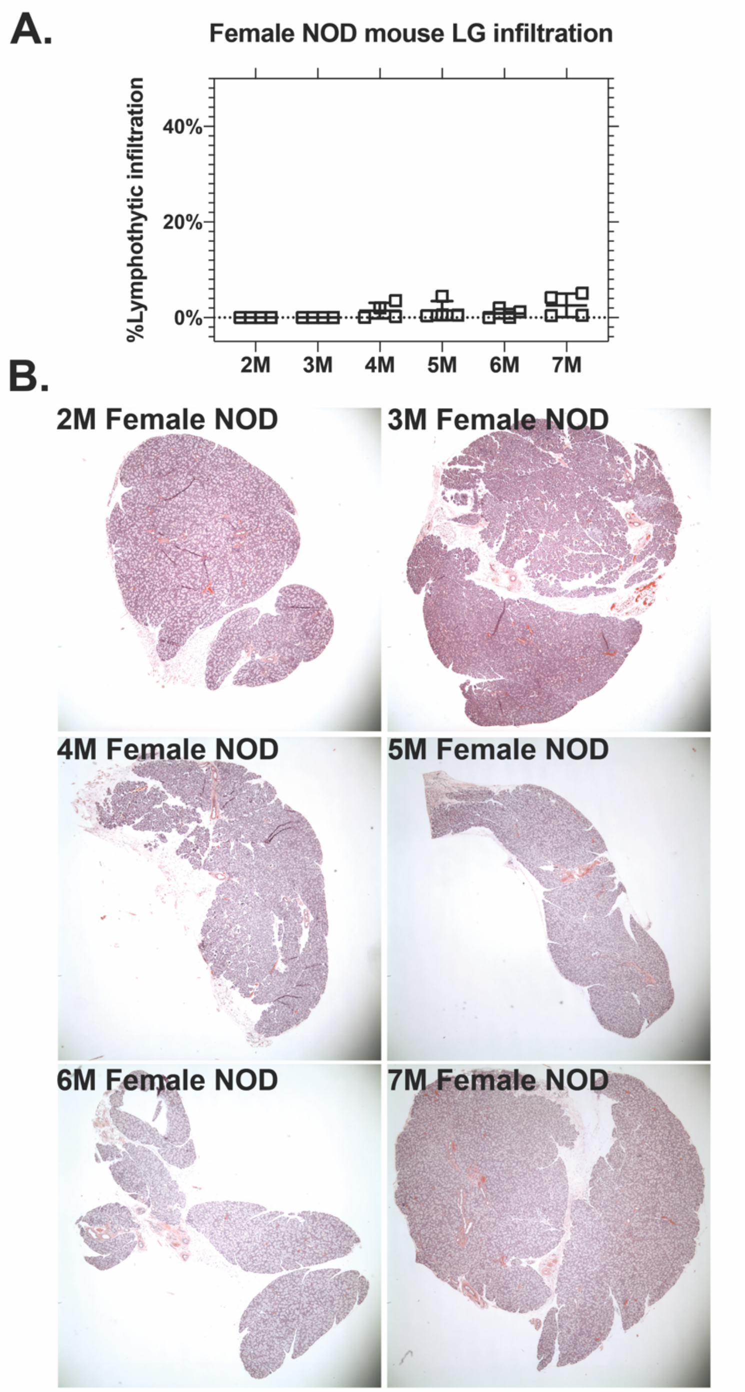

Supplementary Fig. S4. Representative images and quantitative of lymphocytic infiltration in LG of female NOD. 2 to 7 month (M) old female NOD mice (N = 4 mice per group) LGs were sectioned and stained with H&E. **A.** Image quantification from **B**. showed little to no sign of lymphocyte infiltration in female mouse LG. One-way ANOVA with Tukey's multiple comparison test was used to for statistical comparison. No significant difference were detected among age groups. Data represents Mean ± SD.

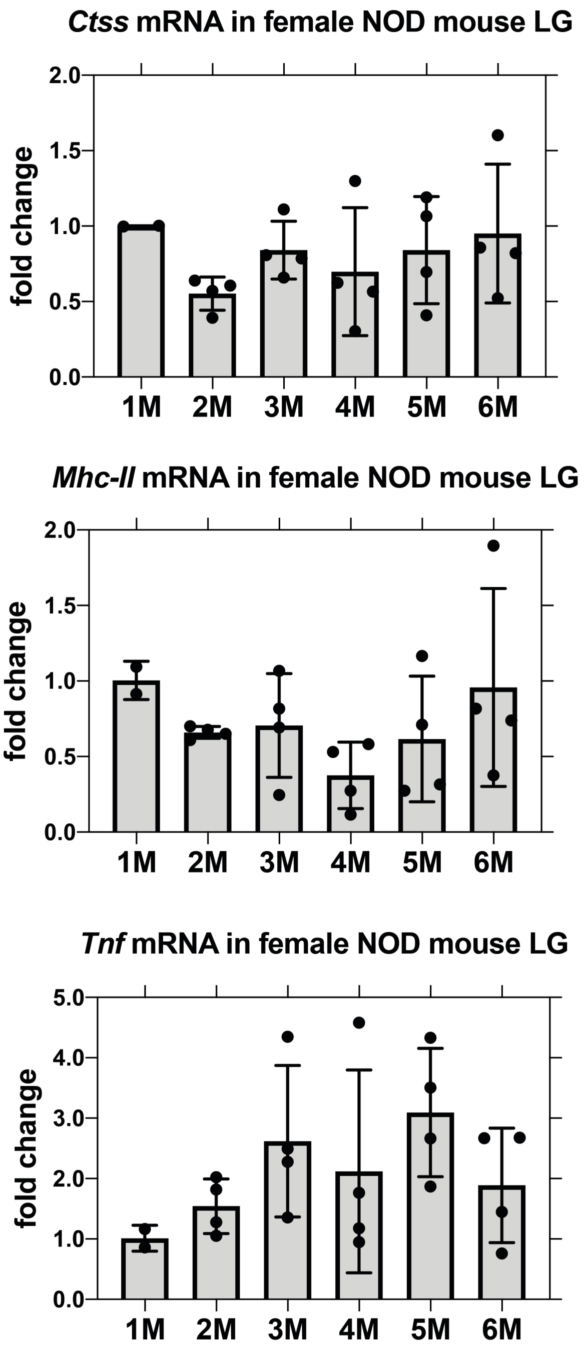

Supplementary Fig. S5. Gene expression of inflammatory indicators Ctss, Mhc-II and Tnf in female NOD mice LG. Gene expression of 1 to 6 month (M) old female NOD mice (N = 4 mice per group) LG were determined by qPCR. One-way ANOVA with Tukey's multiple comparison test was used to for statistical comparison. No significant difference were detected among age groups. Data represents Mean ± SD.

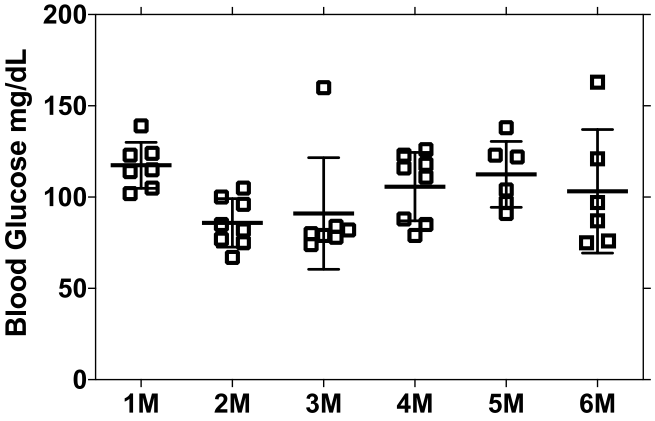

Supplementary Fig. S6. Blood glucose levels in 1 to 6 month (M) old male NOD mice. The levels of glucose were measured in blood to check for diabetes in 1 to 6 M old NOD mice (N=6-8 mice per group). None of the mice developed diabetes (>250 mg/dL) through the experimental endpoint. Data represents Mean ± SD and each point represents a value from one mouse. One-way ANOVA with Tukey's multiple comparison test was used to evaluate significance. No significant changes were observed between the different groups.

Supplementary methods

Cell and tissue processing and confocal fluorescence microscopy: Cells, LG and cornea were processed for immunofluorescence labeling as described previously.[2] The LG and cornea were fixed in 4% paraformaldehyde and 4% sucrose solution in PBS for 3 hr at room temperature. After fixation, tissues were washed with PBS and transferred to a 30% sucrose/PBS solution and left at 4°C overnight. Tissues were embedded in O.C.T compound and frozen on dry ice. O.C.T blocks were sectioned at 5 μm thickness and mounted on glass micro slides (VWR, Radnor, PA). LG cryosections were quenched with NH_4Cl (50 mM) in PBS for 5 min and permeabilised with 0.1% Triton X-100 for 10 min. Sections were blocked with 1% BSA for 1 hr at room temperature, followed by primary antibody and secondary antibody incubations, each for 1 hr at 37°C. PBS washes were applied after each antibody incubation. Corneal cross sections were quenched with NH_4Cl (50 mM) in PBS for 5 min and permeabilised with 0.3% Triton X-100 for 30 min. Corneal sections were blocked with 5% BSA in 0.3% Triton X-100 for 1 hr at room temperature and incubated in primary antibody at 4°C overnight. The sections were washed three times with PBS and incubated with secondary antibody for 1 hr at 37°C. Mouse LGAC were washed twice with PBS after gently removing the culture medium. Cells were then fixed and permeabilised with methanol and acetone (1:1) at -20°C for 10 min, followed by two 5-min PBS washes, and then blocked with 1% BSA at room temperature for 1 hr. The cells were then incubated sequentially with primary antibody and secondary antibody, with three 5-min PBS washes following each incubation. After the final wash, all samples were fixed with

ProLong anti-fade mounting medium and imaged the next day. Images were acquired with a Zeiss LSM 800 with Airyscan. Image quantification was performed using a reported image processing pipeline using python and ImageJ.[3,4]

Blood glucose measurements: As previously described,[5] mice were anesthetised briefly with isoflurane through a nose cone. Peripheral blood was collected by tail nick and measured with Free Style Lite test strips. Mice with blood glucose >250 mg/dl[6] were considered diabetic.

Histology analysis of lymphocytic infiltration in female NOD LG: As previously described,[5,7] LG from female NOD mouse were fixed in 10% neutral buffered formalin solution, embedded in paraffin and cut into 5 µm horizontal sections. The paraffin sections were stained with hematoxylin-eosin (H&E) according to standard procedures. Images of three nonconsecutive whole gland cross sections were obtained with a Nikon 8oi microscope (Melville, NY) equipped with a digital camera. The area of lymphocytic foci occupancy was quantified with ImageJ software by a blinded examiner in each section. The values of three sections were averaged as a single value for lymphocytic infiltration for each mouse.

Primers for qPCR gene expression in female NOD LG: Primers used to analyse gene expression in female NOD mouse LG included *Ctss* (Mm01255859_m1), *Mhc II* (Mm00439216_m1), *Tnf* (Mm00443258_m1), and *Gapdh* (Mm99999915_g1) and were from Applied Biosystems (Grand Island, NY).

References

1 Yu, Y.-R. A. *et al.* A protocol for the comprehensive flow cytometric analysis of immune cells in normal and inflamed murine non-lymphoid tissues. *PLOS ONE* **11**, e0150606, doi:10.1371/journal.pone.0150606 (2016).

2 Meng, Z., Klinngam, W., Edman, M. C. & Hamm-Alvarez, S. F. Interferon-γ treatment in vitro elicits some of the changes in cathepsin S and antigen presentation characteristic of lacrimal glands and corneas from the NOD mouse model of Sjögren's Syndrome. *PLOS ONE* **12**, e0184781, doi:10.1371/journal.pone.0184781 (2017).

3 Tyrpak, D. Corrected-Total-Cell-Fluorescence. doi:https://10.5281/zenodo.3247836 (2019).

4 Gavet, O. & Pines, J. Progressive activation of CyclinB1-Cdk1 coordinates entry to mitosis. *Dev Cell* **18**, 533-543 (2010).

5 Ju, Y. *et al.* NOD and NOR mice exhibit comparable development of lacrimal gland secretory dysfunction but NOD mice have more severe autoimmune dacryoadenitis. *Experimental Eye Research* **176**, 243-251 (2018).

6 King, A. J. F. The use of animal models in diabetes research. *Br J Pharmacol* **166**, 877-894 (2012).

7 Janga, S. R. *et al.* Longitudinal analysis of tear cathepsin S activity levels in male non-obese diabetic mice suggests its potential as an early stage biomarker of Sjögren's Syndrome. *Biomarkers* **24**, 91-102 (2019).
